# Zero Echo Time ^17^O-MRI Reveals Decreased Cerebral Metabolic Rate of Oxygen Consumption in a Murine Model of Amyloidosis

**DOI:** 10.3390/metabo11050263

**Published:** 2021-04-22

**Authors:** Celine Baligand, Olivier Barret, Amélie Tourais, Jean-Baptiste Pérot, Didier Thenadey, Fanny Petit, Géraldine Liot, Marie-Claude Gaillard, Julien Flament, Marc Dhenain, Julien Valette

**Affiliations:** Laboratoire des Maladies Neurodégénératives, MIRCen, CNRS, CEA, Université Paris-Saclay, F-92260 Fontenay-aux-Roses, France; olivier.barret@cea.fr (O.B.); amelie.tourais@cea.fr (A.T.); jean-baptiste.perot@cea.fr (J.-B.P.); didier.thenadey@cea.fr (D.T.); fanny.petit@cea.fr (F.P.); Geraldine.Liot@cea.fr (G.L.); marie-claude.gaillard@cea.fr (M.-C.G.); julien.flament@cea.fr (J.F.); marc.dhenain@cea.fr (M.D.); julien.valette@cea.fr (J.V.)

**Keywords:** cerebral metabolic rate of oxygen, magnetic resonance imaging, oxygen-17, zero echo time, APP/PS1, Alzheimer’s disease, mouse

## Abstract

The cerebral metabolic rate of oxygen consumption (CMRO_2_) is a key metric to investigate the mechanisms involved in neurodegeneration in animal models and evaluate potential new therapies. CMRO_2_ can be measured by direct ^17^O magnetic resonance imaging (^17^O-MRI) of H_2_^17^O signal changes during inhalation of ^17^O-labeled oxygen gas. In this study, we built a simple gas distribution system and used 3D zero echo time (ZTE-)MRI at 11.7 T to measure CMRO_2_ in the APP_swe_/PS1_dE9_ mouse model of amyloidosis. We found that CMRO_2_ was significantly lower in the APP_swe_/PS1_dE9_ brain than in wild-type at 12–14 months. We also estimated cerebral blood flow (CBF) from the post-inhalation washout curve and found no difference between groups. These results suggest that the lower CMRO_2_ observed in APP_swe_/PS1_dE9_ is likely due to metabolism impairment rather than to reduced blood flow. Analysis of the ^17^O-MRI data using different quantification models (linear and 3-phase model) showed that the choice of the model does not affect group comparison results. However, the simplified linear model significantly underestimated the absolute CMRO_2_ values compared to a 3-phase model. This may become of importance when combining several metabolic fluxes measurements to study neuro-metabolic coupling.

## 1. Introduction

Oxidative metabolism is essential to sustain brain’s varying energy needs at rest and during neuronal activation. Therefore, the cerebral metabolic rate of oxygen utilization (CMRO_2_) is a key metric to elucidate brain complex bioenergetic processes in normal conditions and in the context of neurodegenerative pathologies such as Alzheimer’s disease (AD). In AD, accumulation of amyloid beta in the brain has been associated with a defect in mitochondrial function [1] and reduced CMRO_2_ have long been reported in patients [2]. However, whether the defect in oxidative metabolism is the cause the pathological manifestation or rather results from disease progression is still unclear. Being able to investigate this parameter in animal models is critical to evaluate mechanisms involved in neurodegeneration and to evaluate new therapies.

The current gold standard for clinical CMRO_2_ measurements is positron emission tomography (PET) using ^15^O_2_. However, ^15^O-PET presents some major limitations. Because no distinction can be made between hemoglobin-bound ^15^O and metabolic production of H_2_^15^O, a separate exogenous H_2_^15^O intravenous infusion is needed to estimate blood flow and accumulation in tissues. In addition, the half-life of ^15^O is very short (~2 min) and direct access to a cyclotron is required. While it has been used in small animals [3,4], an important constraint remains that the actual spatial resolution is intrinsically limited by the disintegration properties of ^15^O, i.e., the distance that the positron travels prior to annihilation (maximum free path of 8 mm [5]) which, in practice, often limits rodent studies to whole brain measurements [6].

As an alternative to PET, in vivo magnetic resonance (MR) can be used to measure CMRO_2_. While ^1^H-MRI based approaches such as calibrated BOLD offer a high spatial resolution, it requires a transient hyper/hypocapnic challenge [7] and produces whole brain measurements. Moreover, the technique is indirect as BOLD contrast relies on the effect of oxygen on water relaxation properties and is therefore sensitive to several competing physiological effects. ^17^O is the only stable oxygen isotope that can be detected with MR. The low natural abundance of ^17^O (0.037%) makes it particularly suitable for use as a tracer for in vivo isotopic enrichment studies. ^17^O-MRI presents two major advantages over ^15^O-PET: first, the changes in H_2_^17^O signal detected during inhalation of gaseous ^17^O_2_ only originate from metabolically produced water, since gaseous and hemoglobin-bound ^17^O_2_ are “MR-invisible”. There is no need for a separate evaluation of the probe accumulation in the blood, and both experimental procedures and modeling are greatly simplified [8]. Second, and most importantly, there is no intrinsic spatial resolution limit, provided that the detection sensitivity is sufficient. The ^17^O-MRI approach was used to measure CMRO_2_ in human with ultra-short TE imaging at 9.4, 7 and 3 Teslas [8,9,10]. In rodents, optimized spectroscopic MR imaging has been successfully implemented [11,12,13,14]. However, studies using ^17^O imaging remain scarce, which may reflect the challenges of its implementation.

In this work, we implemented an imaging strategy based on zero echo time (ZTE), allowing higher signal-to-noise ratio for the detection of rapidly decaying signal [15], and developed a simple ^17^O_2_ delivery system for the anesthetized animal. We applied ^17^O-ZTE-MRI at 11.7 T to characterize resting state oxidative metabolism in the APP_swe_/PS1_dE9_ mouse model of amyloidosis, one of the hallmarks of Alzheimer’s disease. We processed the results using different quantification models to evaluate the impact of the choice of the parameters on group analysis. Finally, we explored a few possible origins for the observed difference in CMRO_2_ in the APP_swe_/PS1_dE9_ brain by examining differences in cerebral volumes and possible vascular contribution using cerebral blood flow (CBF) estimation.

## 2. Results

### 2.1. Setting Up CMRO_2_ Measurements in the Mouse Brain

**Figure 1:**
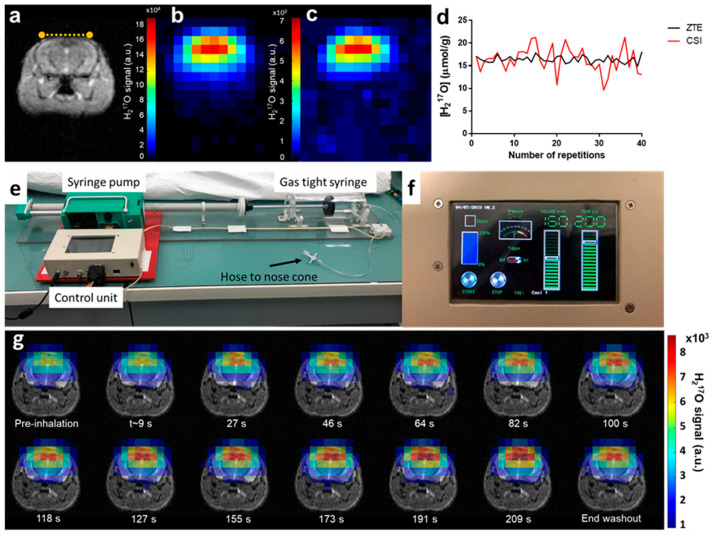
Experimental set-up and in vivo acquisitions. (**a**) Coronal T2 weighted image of a wild-type mouse brain. The yellow dotted line indicates the ^17^O surface coil position on top of the head of the animal. In vivo natural abundance H_2_^17^O images acquired with (**b**) zero echo time (ZTE) and (**c**) chemical shift imaging (CSI) sequences, respectively, demonstrating higher signal-to-noise ratio (SNR) for ZTE. Both images are scaled relative to their respective maximum value. (**d**) Single voxel signal normalized to brain water ^17^O natural abundance displayed over 40 repetitions, showing lower temporal variability for ZTE (black curve) than for CSI (red). (**e**) The ^17^O_2_ delivery system consists of a gas-tight acrylic syringe (500 mL, Hamilton) and a customized syringe pump. The stepper motor is driven by a microcontroller unit (Arduino, Uno R3) and was programmed to allow both manual and MR sequence-triggered activation of the syringe. (**f**) The control unit programmable tactile graphic user interface (4D system, µLCD-43PT) allows prescribing the inhalation protocol parameters (volume, duration and start/stop control). (**g**) The time-course of H_2_^17^O-ZTE images acquired on our Bruker 11.7 T scanner before, during, and after ^17^O inhalation co-registered with the corresponding anatomical image shows incorporation of labeled oxygen in water signal until the end of the inhalation period. Signal decays thereafter and reaches a new steady state, 15 min after the end of the inhalation (end washout).

Spectroscopic imaging has been traditionally used for in vivo ^17^O MR data collection in rodents’ brain [11,12,14,16]. We used 3D free induction decay (FID) chemical shift imaging (CSI) with the shortest achievable echo time (TE = 0.3 ms) as a reference to evaluate the performances of the ZTE approach in a ^17^O natural abundance free water sample and in vivo in the mouse brain. Using identical flip angles and repetition times optimized to maximize signal, we compared the sequences in terms of their signal-to-noise ratio (SNR) and temporal SNR (tSNR) normalized to the square-root of acquisition time and to the effective volume as estimated from the spatial response function (SRF). After spatial filtering (Hamming) of both data sets, ZTE magnitude signal was compared to the integral of the CSI H_2_^17^O peak after application of appropriate line broadening. Phantom data showed that the SNR close to the surface coil was more than two-fold higher with ZTE than with CSI (SNR_ZTE_ = 2.1 min^−1/2^·μL^−1^ and SNRcsi = 0.9 min^−1/2^·μL^−1^). The advantage of ZTE was also evident as a three-fold increase in temporal SNR (tSNR_ZTE_ = 1.7 min^−1/2^·μL^−1^ and tSNR_CSI_ = 0.6 min^−1/2^·μL^−1^). Similar observations were made in vivo for SNR (SNR_ZTE_ = 1.7 min^−1/2^·μL^−1^ and SNR_CSI_ = 0.9 min^−1/2^·μL^−1^; Figure 1b,c), and temporal SNR (tSNR_ZTE_ = 0.9 min^−1/2^·μL^−1^ and tSNR_CSI_ = 0.5 min^−1/2^·μL^−1^, Figure 1d). The optimized ZTE sequence was selected to carry out subsequent 3D CMRO_2_ measurements in mouse brain during inhalation of ^17^O-enriched gas.

To ensure continuous and reproducible delivery of ^17^O-enriched oxygen, we designed a simple breathing circuit (Figure 1e) consisting of a prefilled gas-tight syringe, similar to the implementation by Neveu et al. [17], but with automatic actuation of the plunger by an Arduino controlled stepper motor. Inhalation parameters (volume and duration) were prescribed using the tactile graphic user interface (Figure 1f). A series of 3D ^17^O-ZTE MR images were acquired from each animal’s brain before (5 min), during (3.3 min) and after (15 min) inhalation of ^17^O_2_ with a time resolution of 18 s. Acquisitions provided sufficient SNR to detect the H_2_^17^O signal increase immediately upon inhalation of labeled gas, as illustrated in Figure 1g, showing incorporation of ^17^O_2_ into H_2_^17^O via mitochondrial metabolism.

### 2.2. CMRO_2_ Is Lower in APP_swe_/PS1_dE9_ Than in Wild-Type Mice

**Figure 2:**
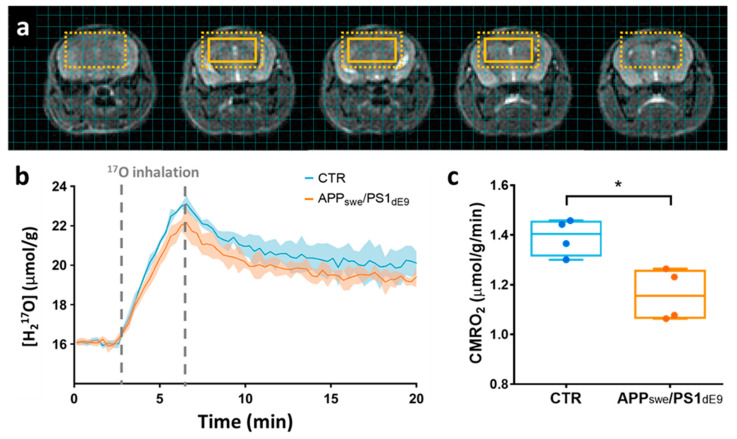
(**a**) Series of 5 contiguous 0.5-mm-thick anatomical images with an overlay showing the extent of the region of interest composed of 8 adjacent voxels. The dotted box indicates the effective resolution after hamming filtering (100 µL). (**b**) Time-courses of H_2_^17^O concentration in APP_swe_/PS1_dE9_ (in orange) and wild-type CTR animals (in blue), presented as group mean ± standard deviations. Grey dotted lines indicate the ^17^O_2_ inhalation period. (**c**) Analysis with a linear model yielded significantly lower CMRO_2_ values (*p* = 0.028) in APP_swe_/PS1_dE9_ (1.16 ± 0.10 μmol/g of tissue/min) compared to wild-type control (1.39 ± 0.07 μmol/g of tissue/min). * *p* < 0.05 (Mann-Whitney’s U test).

We used ZTE-MRI to study the differences in resting state oxygen utilization induced by disease progression in 12–14 month-old APP_swe_/PS1_dE9_ mice compared to age-matched wild-type animals (CTR). To avoid partial volume effect and perform group comparison in this constitutive model of amyloidosis, 8 adjacent voxels (effective volume: 100 μL) were conservatively chosen within the brain of each animal (Figure 2a) for signal averaging. Group averages of the H_2_^17^O signal time curves are shown in Figure 2b. In rodents, where blood circulation time is short, and for short inhalation duration, a simplified linear model can be applied to quantify CMRO_2_ (CMRO_2_ = inhalation slope/(2 α)) [18]. Applying this simplified model to our data collected over the entire 3.3 min inhalation period, and assuming an enrichment fraction α of 0.7 as provided by the vendor, we found CMRO_2_ values of 1.39 ± 0.07 μmol/g of tissue/min in the CTR mice and 1.16 ± 0.10 μmol/g of tissue/min in APP_swe_/PS1_dE9_. This difference between groups was statistically significant (*p* = 0.028, Mann-Whitney’s U test, Figure 2c).

We also evaluated whether the differences of CMRO_2_ between CTR and APP_swe_/PS1_dE9_ brains could be related to differential cerebral perfusion. Post-inhalation H_2_^17^O signal, referred to as the washout phase signal, is mainly driven by local blood perfusion, and the exponential decay rate k_washout_ can be used as an estimator of CBF, as was previously validated in the rat brain [11]. Here, we found no difference in the CBF estimator k_washout_ between groups and values were 0.33 ± 0.03 min^−1^ in CTR and 0.30 ± 0.03 min^−1^ in APP/PS1_dE9_ (*p* = 0.48). Moreover, there was no correlation between CMRO_2_ and k_washout_ in our dataset, suggesting that a rest CBF deficit could not account for the reduced CMRO_2_ in APP_swe_/PS1_dE9_ mice.

### 2.3. Effect of the Choice of the Model on CMRO_2_ Quantification

**Figure 3:**
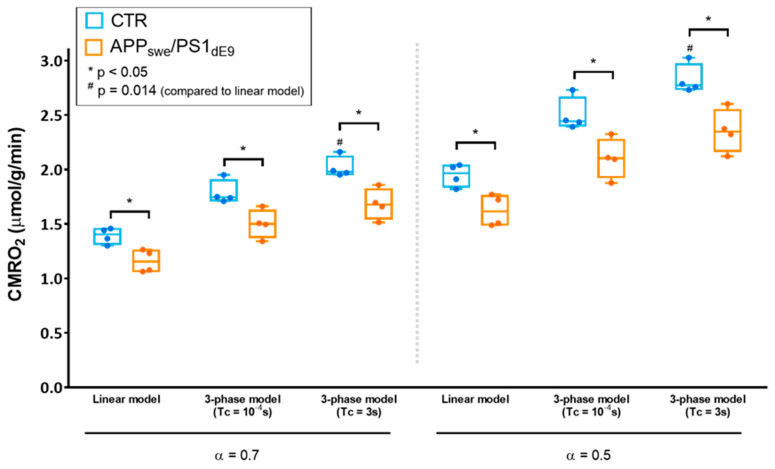
Effect of the choice of the model on CMRO_2_ quantification and group comparison. CMRO_2_ quantified with different models (linear model or 3-phase model) and different sets of parameters (enrichment fraction α = 0.7 or 0.5, blood circulation time Tc → 0 (Tc = 10^−4^ s or Tc = 3 s) are displayed for CTR (in blue) and APP_swe_/PS1_dE9_ (in orange) as min-to-max box and whiskers plots. The grey dotted line separates quantifications ran assuming a ^17^O enrichment fraction of 70 % (α = 0.7) (left side) and 50% (α = 0.5) (right side), thereby accounting for possible contamination of the inhaled mixture with ambient ^16^O_2_ within the nose cone. * indicates significant differences between CTR and APP_swe_/PS1_dE9_ with *p* < 0.05 using a Mann-Whitney’s U test. ^#^ indicates significant differences compared to the results from the linear model using a Friedman test followed by Dunn’s multiple comparison test (*p* = 0.014).

After initial quantification using the simplified linear model, we sought to compare different models of quantification to determine the impact on CMRO_2_ and, most importantly, to assess whether the significant difference in CMRO_2_ between APP_swe_/PS1_dE9_ and control mice depended on the model (Figure 3). We used the 3-phase model initially proposed by Atkinson et al. for quantification of CMRO_2_ in the human brain [8]. In this approach, H_2_^17^O signal is mathematically described before, during, and after ^17^O_2_ inhalation, including the blood circulation time (Tc) as a parameter. Three key parameters can be fitted: CMRO_2_, the rate of labeled water creation in a given voxel, K_G_ and K_L_, rate constants respectively attributed to the “gain” of labeled water due to recirculation and the “loss” due to perfusion. Assuming immediate availability of the labeled oxygen to the tissue (Tc = 0 s), i.e., approaching the hypothesis of the linear model, we found that CMRO_2_ was 1.79 ± 0.11 μmol/g of tissue/min in CTR and 1.50 ± 0.13 μmol/g of tissue/min in APP_swe_/PS1_dE9_ (*p* = 0.028), and the difference between groups was preserved. We then exploited the 3-phase model’s ability to account for a physiological blood circulation rate. Based on literature values [19], we assumed Tc = 3 s. In these conditions, CMRO_2_ was 2.02 ± 0.10 μmol/g of tissue/min in CTR versus 1.68 ± 0.14 μmol/g of tissue/min in APP_swe_/PS1_dE9_ (*p* = 0.028). The fitting procedure also yielded K_G_ and K_L_ values, which were not significantly different between groups. K_G_ was 0.62 ± 0.18 min^−1^ in CTR and 0.45 ± 0.06 min^−1^ in APP_swe_/PS1_dE9_ (*p* = 0.2), and K_L_ was 0.39 ± 0.04 min^−1^ in CTR and 0.36 ± 0.06 min^−1^ in APP_swe_/PS1_dE9_ (*p* = 0.7). The same analyses were then computed assuming that the effective ^17^O enrichment fraction α of the inhaled mixture was lower than that of the gas contained in the prefilled syringe due to contamination by ambient air. We set α = 0.5 and obtained CMRO_2_ values of 1.95 ± 0.10 μmol/g of tissue/min in CTR and 1.62 ± 0.14 μmol/g of tissue/min in APP_swe_/PS1_dE9_ (*p* = 0.028) with the linear model. The complete model with Tc = 3 s yielded CMRO_2_ values of 2.83 ± 0.14 μmol/g of tissue/min in CTR and 2.36 ± 0.20 μmol/g of tissue/min in APP_swe_/PS1_dE9_ (*p* = 0.028). K_G_ was 0.87 ± 0.25 min^−1^ in CTR and 0.63 ± 0.09 min^−1^ in APP_swe_/PS1_dE9_ (*p* = 0.2), and K_L_ was 0.40 ± 0.04 min^−1^ in CTR and 0.36 ± 0.06 min^−1^ in APP_swe_/PS1_dE9_ (*p* = 0.7). While the difference between groups was preserved regardless of the model used, an analysis of variance (ANOVA) run on the CTR data at either α = 0.7 or α = 0.5 showed that the choice of the model impacted significantly the absolute value computed for CMRO_2_ (*p* = 0.0015, Friedman test). Specifically, in both cases, Dunn’multiple comparison test showed significant differences between the linear model and the 3-phase model with Tc = 3 s (*p* = 0.014).

## 3. Discussion

^17^O-MRI based measurement of oxygen metabolism has evidently raised increasing interest over the past 30 years [20,21]. However, it is not yet widely used, mostly due to the cost and complexity of implementation. The current report shows the first implementation of ZTE MR imaging in mice for ^17^O data collection and CMRO_2_ measurement during an enriched ^17^O_2_ gas inhalation experiment. We found that using a vendor-supplied ZTE sequence was advantageous over the standard FID CSI, yielding a two-fold increase in SNR. Combined with the simple inhalation set-up proposed in this study, this makes the technique readily reproducible in other preclinical imaging centers, and may promote the use of ^17^O-MRI for the study of neurodegenerative disorders.

An important application of the technique and a major finding of this study was the significantly lower CMRO_2_ in APP_swe_/PS1_dE9_ mice compared to age-matched wild-type. We are confident that this difference in CMRO_2_ is a robust result because:

(1) It does not depend on the model used for quantification as shown in Section 2.3,

(2) Relative ventricle/brain volume differences between groups can be ruled out as possible bias. Indeed, high-resolution T2-weigthed MRI were performed on each animal and automated segmentation showed no significant difference in total brain volume (WT: 467 ± 18 mL vs. APP/PS1: 484 ± 12 mL, *p* = 0.16) and ventricles volume (WT: 4.27 ± 0.12 mL vs. APP/PS1: 4.49 ± 0.18 mL, *p* = 0.25).

Metabolic disturbances are well documented in Alzheimer’s patients as glucose hypometabolism in brain regions typically affected by the pathology [22,23,24] and oxygen metabolism perturbation [2]. Previous studies, mostly performed in vitro, have shown that amyloidosis disturbed mitochondrial function and citric acid cycle enzyme activities [25], which would translate into impaired oxygen metabolism. In a different mouse model (Arcβ mice), Ni et al. [26] inferred CMRO_2_ from CBF and oxygen extraction fraction and found that it was decreased in aged Arcβ mice. While lower CMRO_2_ are not unexpected in the aged APP_swe_/PS1_de9_ mice where amyloidosis begins around 4 months of age [27], in vivo data directly linking Aβ accumulation and CMRO_2_ are largely lacking. The ^17^O-MRI approach implemented in our study allows for direct and non-invasive measurement of CMRO_2_ and will enable longitudinal studies in Alzheimer’s disease models to further investigate the origins of CMRO_2_ alterations.

In addition to CMRO_2_ quantification, the ^17^O_2_ inhalation experiments provide an estimation of CBF through the H_2_O^17^ signal exponential decay rate (k_washout_) measured during the washout period. In rats, it was shown that k_washout_ can be converted into a quantitative measure of CBF using the empirically determined conversion factor of 1.86 [16]. A similar calibration study would be required to determine this coefficient in mice in our experimental conditions to allow CBF quantification. Nonetheless, k_washout_ can be directly used as an index of resting state CBF for group comparison. While we cannot exclude that vascular function and reactivity is altered in the APP_swe_/PS1_dE9_ model, which is known to display amyloid angiopathy [27], k_washout_ was not significantly affected in our group of mice. This suggests that the lower resting state CMRO_2_ does not simply result from restricted access to oxygen due to limited blood flow, but rather indicates altered mitochondrial function. This is consistent with previous studies in aged APP/PS1 that have reported decreased COX and SDH activity [28], as well as lower PGC1α and Tfam protein levels [29], suggesting a mitochondrial impairment in this model. Resting state CBF might nonetheless be regionally impaired in APP_swe_/PS1_dE9_, which would not necessarily be captured by our analysis over 8 voxels and/or might not reach significance given our small sample size. In the same model, a previous study reported a decrease in cortex CBF [30]. Future studies should include H_2_^17^O bolus injection experiments to calibrate CBF quantification using the post inhalation phase, or, preferably, arterial spin labeling MRI measurements to obtain higher resolution CBF maps in these mice.

The CMRO_2_ values found in wild-type animals were lower than previously published results using the same quantification model (linear model). For instance, Zhu et al. [16] reported a CMRO_2_ value of 2.63 ± 0.16, Cui et al. [11] of 2.6 ± 0.4 μmol/g/min, and Lou et al. [12] consistently found 2.72 ± 0.46 μmol/g/min. There are several possible explanations to this difference. First, the wild-type mice in our study were scanned at ~12 months of age in order to age-match the late stage APP_swe_/PS1_dE9_ animals, whereas most previous ^17^O-MRI studies were carried out in ~3 month-old animals [11,12]. In mice, there are conflicting reports of either no change [26,31] or even an increase in CMRO_2_ [32] with age. However, these studies were performed using indirect estimates of CMRO_2_. In humans, several ^15^O-PET studies have shown that resting brain oxygen metabolism was decreased in elderly [33,34,35,36]. It cannot be excluded that the lower CMRO_2_ could be an effect of age in our animals. Another possible contribution to our lower CMRO_2_ values is the choice of the anesthetic. In previous studies, isoflurane (1.2 to 2%) [11,12] or ketamine/xylazine infusion [16] were used for MR acquisitions. Here, we used medetomidine, which is known to have a vasoconstrictor effect [37] and to induce lower resting state perfusion [38], as opposed to isoflurane, which is a vasodilatory agent [39]. Medetomidine was also shown to alter glucose metabolism [40]. Altogether, these effects of anesthesia may alter oxygen availability and CMRO_2_. Lastly, it cannot be excluded that, with our gas distribution set-up and nose cone, a fraction of what the animal inhaled came from surrounding air containing ^16^O_2_, thereby decreasing the ^17^O enrichment fraction of the mixture inhaled to an unknown value. To evaluate this effect, we quantified the results using either α = 0.7 as administered or α = 0.5, assuming that 20% of the gas mixture contained ^16^O_2_. Resulting CMRO_2_ values were higher when assuming α = 0.5, however not reaching the 2.7 µmol/min/g previously reported [11]. It is likely that a combination of these factors explains our lower values in wild type.

The statistically significant group differences found in this study were preserved regardless of the model used for quantification. However, our results comparing the simplified linear model to the 3-phase model with different sets of parameters showed their impact on absolute values. The linear model may lead to an underestimation of CMRO_2_ compared to the 3-phase model. Indeed, the assumption of a very rapid blood circulation time underlying the linear model may be challenged in certain pathological conditions where cardiac function and/or local blood flow is affected, increasing Tc. Moreover, the validity of the linear model is bound to short inhalation times, which may be suboptimal in the context a slow oxygen metabolism such as in the diseased brain or in other organs like muscles [13], where H_2_^17^O enrichment is slower and requires longer inhalation times. Absolute quantification becomes crucial when combining the measurements of different fluxes (e.g., metabolic rate of glucose, ATP production rate or CBF) to precisely study neuro-metabolic coupling and infer the oxygen-glucose index similar to what is done in PET studies [41,42], or to infer the oxygen extraction fraction from CBF and CMRO_2_ [16]. Because blood flow and circulation time may be differentially affected by the anesthesia regime, or may simply be altered by the pathology, preliminary experiments may be recommended to determine Tc and select the appropriate model prior to running a preclinical study.

## 4. Materials and Methods

### 4.1. Animals and Preparation

All experimental protocols were reviewed and approved by the local ethics committee and submitted to the French Ministry of Higher Education, Research, and Innovation (approval: APAFIS#21333-2019062611). They were performed in a facility authorized by local authorities (authorization #D9203202), in strict accordance with recommendations of the European Union (2010-63/EEC). Mice were housed in standard conditions (12-h light-dark cycle, temperature: 22 ± 1 °C and humidity: 50%) with ad libitum access to food (Altromin 1310) and water.

MR experiments were performed in a group of APP_swe_/PS1_dE9_ mice (*n* = 4, 24.4 ± 1.4 g, age = 13 ± 1 months) and a group of wild-type littermates (CTR, *n* = 4, 24.8 ± 2.1 g, age = 11.6 ± 0.3 months). The mice co-express human APP with the Swedish double mutation (KM670/671NL) and human PS1 deleted in exon 9 under the control of the mouse prion protein promoter. These mice develop characteristic β-amyloid plaques and angiopathy in cortex and hippocampus starting at 4 months and increasing with age, as previously described [27,43]. High amyloid load was seen in the mice involved in our study by immunohistochemistry (see Supplementary Figure S1). Before each scan, animals were initially anesthetized with isoflurane mixed in medical air (3% for induction, 1.5% for maintenance) and a catheter was inserted in the tail vein. Mice were then placed prone on a water-heated bed equipped with temperature and breathing rate monitoring, and anesthesia was switched from isoflurane to medetomidine. Mice received an initial intravenous bolus of medetomidine (0.1 mg/kg domitor^®^, Vetoquinol) and an infusion was immediately started for maintenance (0.2 mg/kg/h). Isoflurane was progressively decreased to 0% within 10 min. Once placed in the magnet and maintained under medetomidine anesthesia only, mice were supplied with non-labeled O_2_ through a nose cone. The CMRO_2_ measurement protocol started within 45 min from the first anesthesia, when physiological parameters were stable (~120–140 breath/min, ~36.5 °C body temperature).

### 4.2. MR Methods

MR experiments were performed on a horizontal 11.7 T scanner (Bruker, Ettlingen, Germany) interfaced with Paravision 6.0.1. A 72-mm diameter ^1^H quadrature volume coil (Bruker, Ettlingen, Germany) and a custom-built 10-mm diameter ^17^O surface coil were used. After automatic adjustments including ^1^H RF calibration, global shimming and frequency adjustment, a fast low angle shot (FLASH) image was acquired for localization and shim volume preselection (TR/TE = 195/2.3 ms, field of view (FOV) = 4 cm × 4 cm, matrix size = 256 × 256). It was followed by a 3D B0 field map (TR/TE1/TE2 = 20/1.4/5.4 ms, FOV = 3 cm × 3 cm × 3 cm, matrix size = 64 × 64 × 64, NA = 8) with a signal-to-noise threshold of 10 for reconstruction. A ^1^H PRESS scan (2048 points, 4.5 mm^3^) was used to evaluate the shim quality over the entire brain, before and after the shim calculation procedure was applied (MAPSHIM). The resulting full width at half maximum for ^1^H ranged between 45 and 55 Hz in vivo. In the CTR and APP_swe_/PS1_dE9_ groups, RARE images were acquired (TR/TE = 3000/30 ms, RARE factor 8; FOV: 20 × 20 mm^2^; matrix size: 192 × 192; slice thickness: 0.5 mm) so that total brain and ventricles volumes were determined by automated segmentation using an in-house developed python library (https://sammba-mri.github.io/, accessed on 1 November 2018).

#### 4.2.1. SNR Comparison Experiments

To maximize sensitivity and overcome the short T2* expected for ^17^O, in vivo ^17^O-MRI was acquired using a hard pulse ZTE approach. We first compared the 3D ZTE performance to that of a standard 3D CSI on natural abundance water phantom and in vivo. For both sequences, RF pulse (5-μs broad pulse, 100 W) and TR (1.8 ms) were kept identical. ***CSI acquisitions:*** For 3D CSI, echo time was set to TE = 0.3 ms, the minimum achievable TE value in our settings. The field of view was 24 × 24 × 24 mm^3^, the matrix size was 16 × 16 × 16, the number of complex points was 128 with a dwell time of 8.4 µs, and the number of averages was 3. Successive CSI blocks were acquired with 4 averages in 24.8 s each, and repeated 40 times. ***CSI post-processing***: 3D complex data were exported to Matlab (The Mathworks Inc., Matlab, R2015b) and filtered with a Hamming window. FIDs were then zero filled to 256 points and a line broadening of 6000 Hz was applied to minimize the FID truncation artifact due to the short acquisition time (1 ms). After Fourier transform and spectral rephasing, voxelwise integration of the real part over 3720 Hz (9 points) was performed to generate CSI images. ***ZTE imaging acquisitions:*** ZTE cartesian matrix size was 32 × 32 × 32 for a field of view of 48 × 48 × 48 mm^3^, which was achieved with 3310 radial spokes, each acquired in 0.88 ms. Successive ZTE blocks were acquired with 4 averages in 24.8 s, and repeated 40 times. ***ZTE post-processing***: Initial reconstruction was performed in Paravision 6.0.1 (Bruker, Ettlingen, Germany), including reggriding of the ZTE-MRI radial k-space onto a Cartesian k-space using a Kaiser-Bessel kernel, density compensation and apodization correction. Complex Cartesian data were exported to Matlab and filtered with a Hamming window. ***SNR calculation***: Spatial response functions were calculated accounting for the Hamming filter for CSI, resulting in an effective volume V_eff_ = 2.84 mm^3^ for individual voxels, and for Hamming filter and T2* relaxation for ZTE acquisition, with a T2* of 1.3 ms in vivo (resulting in V_eff_ = 3.1 mm^3^), and 3.2 ms in free water (resulting in V_eff_ = 2.9 mm^3^), as estimated from unlocalized single pulse acquisitions. *SNR* was calculated for a pixel located close to the coil for ZTE and CSI images (averaged over the 40 repetitions) as:

(1) $$SNR=\frac{signal}{SD\left(noise\right)\times V_{eff}\times \sqrt{block\;acquisition\;time\;xNR}}$$

where noise is a 6 × 6 pixels^2^ region of interest outside of the object.

The temporal *SNR* was calculated as:

(2) $$tSNR=\frac{\sum_{n=1}^{NR}signal_{n}/NR}{SD\left(signal_{n,\;n:1\to NR}\right)\times V_{eff}\times \sqrt{block\;acquisition\;time\;}}$$

#### 4.2.2. ^17^O_2_ Inhalation Experiments

Inhalation experiments were conducted in CTR and APP_swe_/PS1_dE9_ mice with the same parameters as described above for SNR comparison except for TR = 1.3 ms (chosen to further maximize in vivo SNR), yielding an individual scan time of 18.2 s. A series of ZTE-MRI were continuously acquired before (5 min), during (200 s), and after (15 min) inhalation of 70%-enriched ^17^O_2_ (Nukem Isotopes, GmbH, Alzenau, Germany). To that effect, the breathing circuit connected to the nose cone was transiently switched from ^16^O_2_ delivery to a home-built gas delivery system (Figure 1e,f) that started to deliver 160 mL ^17^O-enriched O_2_ over 200 s. The ^17^O_2_ delivery system consisted of a 500 mL gas-tight acrylic syringe (Super Syringe Model S0500 TLL, PTFE Luer Lock, Hamilton France SARL, Villebon-sur-Yvette, France) and a modified infusion pump, which stepper motor was driven by an Arduino Uno R3 board (https://www.arduino.cc/en/main/software, accessed on 1 August 2019). A programmable tactile graphic user interface (µLCD-43PT, 4D Systems, Minchinbury, Australia) allowed prescribing the inhalation protocol parameters (volume, duration and start/stop control). Data were processed using the same steps as for the SNR comparison experiments, described in Section 4.2.1 above.

### 4.3. CMRO_2_ Calculation

First, each series of filtered H_2_^17^O ZTE images was normalized to baseline values in a pixel-wise manner and converted to absolute H_2_^17^O concentration at baseline (D). Assuming a natural abundance of 20.35 μmol of H_2_^17^O per g of water and a water/brain partition coefficient β_brain_ of 0.79 [18], the value D is equal to 16.07 μmol/g of brain tissue and CMRO_2_ results can be expressed in μmol of O_2_/g of brain tissue/min. CMRO_2_ quantification was achieved using two different approaches: (1) the 3-phase metabolic model proposed by Atkinson et al. [8] and (2) the simplified linear model proposed and validated by Zhang et al. [18] in rodents. All models and fitting procedures were implemented in Matlab.

#### 4.3.1. Three-Phase Model

The 3-phase metabolic model describes H_2_^17^O MR signal before, during, and after ^17^O_2_ inhalation. H_2_^17^O signal changes result from the combination of three parallel processes: the local metabolic production of H_2_^17^O in the tissue, the efflux H_2_^17^O through diffusion and perfusion (washout), and the entry of H_2_^17^O metabolically produced outside of the voxel (recirculation from the entire body). H_2_^17^O MR signal can be described by the following equation:

(3) dMbrainH2O17(t)=2 · CMRO2 · AO17(t)−KL · MbrainH2O17(t)+KG · BH2O17(t)

where AO17(t) is the fraction of arterial ^17^O_2_ in excess of natural abundance, BH2O17 is the relative amount of H_2_^17^O in the blood in excess of natural abundance, *K_L_* is the rate constant reflecting the loss of H_2_^17^O mostly due to perfusion and *K_G_* is the rate constant reflecting the gain of H_2_^17^O, mostly due to recirculation of blood containing labeled water produced throughout the body. The details of this final expression can be found in Atkinson et al. [8]. Atkinson’s formalism explicitly includes the circulation time (Tc) as a modulator of the blood enrichment fraction and recirculation contribution. However, it should be noted that, restricting the equation to the inhalation period and assuming that the arterial ^17^O_2_ enrichment is immediate with a blood saturation of 100%, it is equivalent to the linear model proposed by Zhu et al. [14] and described in the next section.

#### 4.3.2. Simplified Model

A simplified model has been proposed by Zhang et al. [18] based on their earlier work [14]. The model proposed by Zhu et al. is essentially equivalent to the mathematical description by Atkinson et al. but restricted to the inhalation phase, and with the extra assumption that all inhaled labeled oxygen is immediately available to the cells, translating into Tc being close to 0. This assumption is commonly accepted in small animal models where blood circulation is very rapid, although values ranging from 3 to 4.5 s can be found in the literature [19,44]. This model was further simplified by Zhang et al. [18] to a linear relationship between the slope of H_2_^17^O signal changes during inhalation and has been empirically validated in vivo in rats. As a result, *CMRO*_2_ can be calculated as:

(4) $$CMRO_{2}=\frac{a}{2\alpha },$$

where *a* is the slope of the linear fit of the inhalation phase, and *α* is the ^17^O enrichment fraction of the delivered gas.

### 4.4. CBF Estimation

We used the mono-exponential decay rate of post-inhalation H_2_^17^O signal as an estimate of CBF, as was described and validated by Zhu et al. [16] in rats. The following equation was fitted to the data:

(5) [H2O17]= k1×e−kwashout ×t+k2 ,

where k_1_ and k_2_ are constants, and *k_washout_* is proportional to CBF.

### 4.5. Immunohistochemistry

The 4% paraformaldehyde post-fixed hemispheres were cryoprotected using 15% and 30% sucrose solutions. Series of brain coronal sections (40-µm-thick) were cut on a sliding freezing microtome (SM2400, Leica Microsytem). The floating histological serial sections were preserved in a storage solution (30% glycerol, 30% ethylene glycol, 30% distilled water, and 10% phosphate buffer) at −20 °C until use. Serial sections of the entire brain were stained for the evaluation of Aβ pathology (4G8 immunohistochemistry). Brain sections were rinsed with PBS, pre-treated with 70% formic acid for 3 min and then incubated in 0.3% hydrogen peroxide for 20 min. Sections were then blocked with PBS-0.2% Triton (Triton X-100, Sigma, St. Louis, MO, USA) and 4.5% normal goat serum (NGS) for 30 min before overnight incubation with biotinylated-4G8 at 4 °C (1:500; Biolegend Covance #SIGNET-39240, monoclonal). The sections stained were rinsed with PBS and then incubated with ABC Vectastain (Vector Labs) before diaminobenzidine tetrahydrochloride (DAB) revelation (DAB SK4100 kit, Vector Labs). Brain sections were observed using a Leica DM6000 microscope equipped with a digital color camera (MicroFireTM, Optronics, Goleta, CA, USA).

### 4.6. Statistical Analysis

All statistical analysis were performed using GraphPad Prism version 6.07 for Windows (GraphPad Software, San Diego, CA, USA, www.graphpad.com, accessed on 22 April 2021). Group comparison were tested using Mann-Whitney’s U test and statistical significance was set to *p* < 0.05. Linear correlations were tested using a Pearson coefficient r and and statistical significance of the correlation was set to *p* < 0.05. Comparison of the results from the different models in the CTR data set were performed using non-parametric ANOVA (Friedman) and Dunn’s test for multiple comparisons.

## Data Availability

All data are available within the manuscript.

## Supplementary Materials

The following are available online at https://www.mdpi.com/article/10.3390/metabo11050263/s1, Figure S1: Detection of amyloid-β by 4G8 immunostaining in the brain of 14 month-old APP_swe_/PS1_dE9_ mice and not in CTR.
