# Supplementary material for: Zero Echo Time 17O-MRI Reveals Decreased Cerebral Metabolic Rate of Oxygen Consumption in a Murine Model of Amyloidosis
Source: Metabolites. 2021 Apr 22;11(5):263. doi: 10.3390/metabo11050263 (PMC8145383; doi:10.3390/metabo11050263)
Supplement: Supplementary file 1 [file metabolites-11-00263-s001.zip › metabolites-1172259-supplementary.pdf]

Supplementary Materials

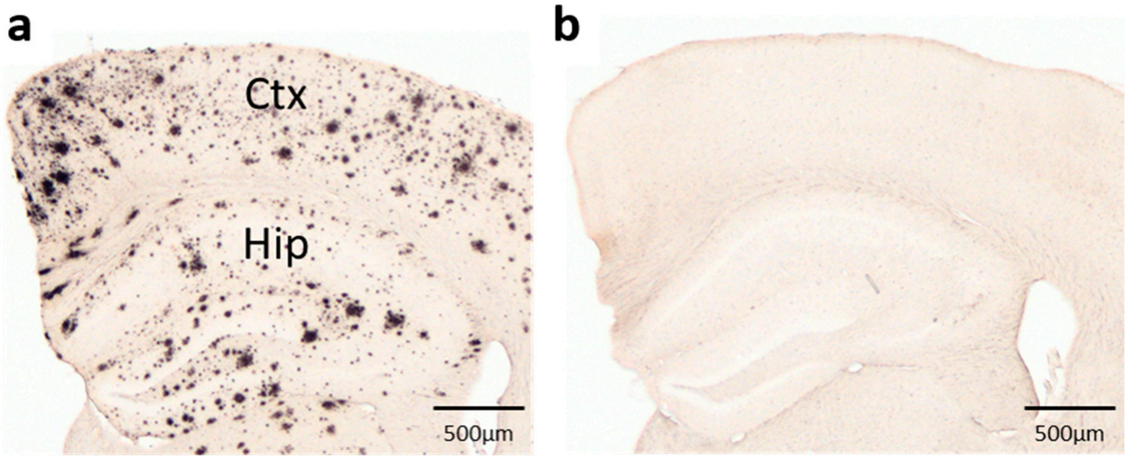

Figure S1. Detection of amyloid-β by 4G8 immunostaining in the brain of 14 month-old $APP_{swe}/PS1_{dE9}$ (**a**) mice and not in CTR (**b**). Ctx: cortex, Hip: hippocampus.
